# Alternative Dosing of Eltrombopag for the Treatment of Chronic, Steroid-Refractory Immune Thrombocytopenia

**DOI:** 10.7759/cureus.63203

**Published:** 2024-06-26

**Authors:** Salman Syed, Hadassah Stein, Marc Ganz, Daniel Miller, Garry Lachhar, Harinder Sawhney

**Affiliations:** 1 Internal Medicine, Northwell Health, New York City, USA; 2 Radiology, Northwell Health, New York City, USA; 3 Public Health Sciences, State University of New York Downstate Health Sciences University, Brooklyn, USA; 4 Internal Medicine, Icahn School of Medicine at Mount Sinai, Queens Hospital Center, Queens, USA; 5 Medicine, Northwell Health, New York City, USA

**Keywords:** eltrombopag, alternative dosing, purpura, immune thrombocytopenia, pharmacology

## Abstract

Idiopathic thrombocytopenic purpura (ITP) presents challenges in management, particularly in cases refractory to corticosteroids. Thrombopoietin receptor (TPO-R) agonists, such as eltrombopag, offer alternative therapeutic options. We report a case of a 72-year-old South Asian male with steroid-refractory chronic ITP who achieved a favorable response with biweekly eltrombopag dosing. Platelet response was comparable to daily dosing, suggesting the efficacy of less frequent administration schedules. This approach may enhance patient adherence and reduce treatment-related financial burdens. Biweekly eltrombopag dosing presents a promising alternative for chronic ITP management, warranting further investigation.

## Introduction

Idiopathic thrombocytopenic purpura (ITP) is a condition where patients have low platelet counts without apparent cause; it is a diagnosis of exclusion. Given that most cases of ITP seem to have an autoimmune component, with antibodies recognizing self-antigens on the platelet surface, corticosteroids are the standard first-line treatment [1]. Thrombopoietin receptor (TPO-R) agonists are trialed in steroid-refractory cases.

TPO-R agonists spare patients from splenectomy or the immunosuppressive effects of rituximab, which are both possible management strategies for steroid-refractory ITP. The standard dosing protocol for a TPO-R agonist is 50 mg daily, with a modified dosing protocol of 25 mg daily for people of East Asian origin [2]. Daily dosing can be cumbersome and costly for patients. However, one such agent, eltrombopag, has an extended half-life of 26-35 hours, which lends itself to less frequent dosing protocols [3]. We present a case of a 72-year-old man of South Asian origin with steroid-refractory chronic ITP who achieved an acceptable response with twice-weekly dosing of eltrombopag. In fact, the platelet response rate was comparable to that expected with once-daily dosing. Less frequent dosing protocols can increase patient compliance, reduce adverse effects, and minimize patient financial burdens.

## Case presentation

A 72-year-old South Asian male with a past medical history of coronary artery disease with two stents and hypertension on atorvastatin, aspirin, and atenolol was incidentally found to have a platelet count of 28,000 on routine blood work. The patient endorsed feeling fatigued but denied any history of bleeding gums, blood per rectum or in urine, blisters in the mouth, or vision changes. He reported that his last blood work was done when he underwent coronary artery stenting a couple of years prior. All results were normal at that time. He was a former smoker with a 13-pack-year history but quit smoking 20 years ago. He received two COVID mRNA vaccines during the pandemic and never contracted the virus to his knowledge. There was no known family history of hematological disorders. Physical exam was negative for petechiae, purpura, mucocutaneous bleeding, or splenomegaly.

On labwork, white blood count, red blood count, and Hb were within normal limits. Hematocrit was slightly elevated at 53.1 (normal reference 40.1-49). Absolute neutrophils were somewhat elevated at 7,936 cells/microliter (normal reference 2,500-7,000 cells/microliter).

First, the patient’s aspirin and atorvastatin, which have been implicated in thrombocytopenia, were held. When no significant improvements in platelet numbers were seen, the patient underwent a more extensive workup. Given that he was native to South Asia, where dengue is endemic, serology for dengue fever was drawn. He was also tested for hepatitis B, C, HIV, Helicobacter pylori (H. pylori), and lupus, which are all associated with thrombocytopenia. All tests returned negative. While Coomb’s testing is often part of the standard thrombocytopenia workup to rule out combined hemolytic anemia and immune thrombocytopenia or “Evan’s syndrome,” it was omitted in this case because the patient did not have anemia.

According to current guidelines, in adults, thrombocytopenia is only treated if counts are below 25,000 to 30,000/MCL or if patients have symptomatic bleeding [4]. Given the patient’s platelet count was 28,000, the decision was made to proceed with treatment. According to the standard treatment protocol for idiopathic cases, the patient was started on oral dexamethasone 40 mg daily for four days without significant change. The dose was titrated up to 1 mg/kg prednisone for two weeks. Platelet numbers initially increased but were not sustained, dropping to 30,000 once the steroid course was complete.

The patient was then placed on the TPO-R agonist, eltrombopag. The standard dosing of eltrombopag is 50 mg once daily, with reduced dosing of 25 mg daily for patients of East Asian origin or those with moderate or severe liver insufficiency [3]. However, the patient was trialed on a reduced-frequency, biweekly dosing schedule with an effective platelet response as outlined in Table 1.

**Table 1:** Trends in platelet counts at the end of each week of therapy

Platelet Response With 50 mg Biweekly Eltrombopag Dosing	
Time	Platelet Count (In thousands)
Week 1	75
Week 2	150
Week 3	100
Week 4	125
Week 5	150
Week 6	175
Week 7	180
Week 8	170
Platelet counts remained stable at the time of case publication	
Normal Platelet Reference Range: 150,000 to 400,000	

Other than mild transient hepatotoxicity on labwork, the patient did not experience any significant side effects. The patient was able to resume aspirin and atorvastatin.

## Discussion

**Figure 1:**
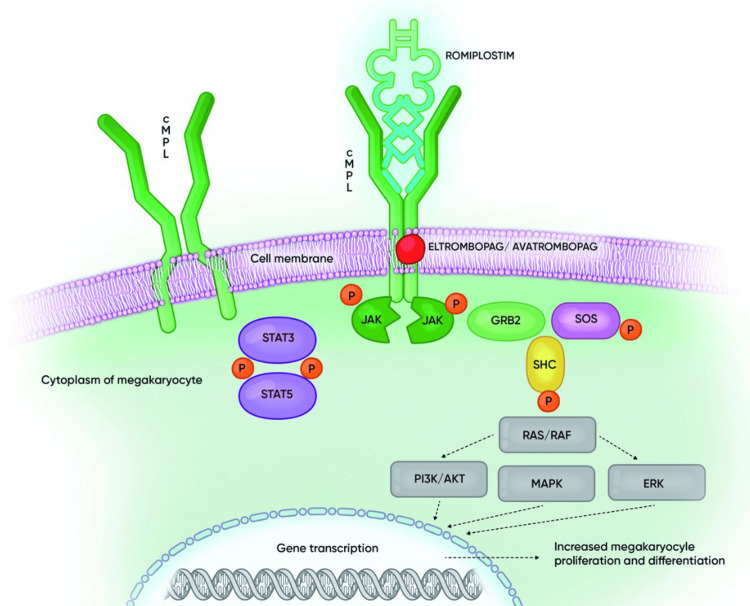
Mechanism of action of eltrombopag and romiplostim GRB2: growth factor receptor-binding protein, JAK: Janus kinase, MAPK: mitogen-activated protein kinase, P: phosphorylation, RAF: rapidly accelerated fibrosarcoma kinase, RAS: rat sarcoma GTPase, SHC: Src homology collagen protein; STAT: signal transducer and activator of transcription; PI3K: phosphatidylinositol 3-kinases, ERK: extracellular-signal-regulated kinase Image used with permission from Ghanima, 2018; Permission nr. 005/2024 [5]

ITP is a condition where patients have unexplained low platelet counts. The pathophysiology is not fully understood, but it is thought to be an autoimmune process. One theory is that when platelets become coated with IgG autoantibodies, they are cleared in the liver and spleen. Most people will then have a compensatory increase in platelet production. In some, platelet production appears to be impaired by possibly intramedullary infiltration and destruction by those antibodies [1]. TPO-R agonists, such as eltrombopag, are used in steroid-refractory ITP. The mechanism of action of eltrombopag is illustrated in Figure 1. 

The EXTEND study in 2013 followed 299 patients treated with eltrombopag for up to three years. Responses were seen in patients with and without splenectomy at 80% and 88%, respectively. With continued administration of eltrombopag, responses were maintained for approximately 70% of the study duration [6]. A follow-up study published in 2017 reported that 259 of 302 study participants, or 86%, had a platelet count >50,000/microliters at least once, with 133 of 257, or 52% of patients achieving sustained response of 25 weeks or more. Intolerable adverse events leading to withdrawal included hepatobiliary changes, cataracts, thrombosis, headache, and myelofibrosis, which occurred in 41% or 14% of participants [6].

The median duration of eltrombopag treatment is 2.4 years, with 25 mg to 50 mg daily [7]. The long duration of treatment and frequent dosing increases the risk for toxicity and places financial strain on the patient. In this case report, we observed a patient with chronic ITP who received treatment with the intermittent eltrombopag dosage protocol. We evaluated the drug's safety and efficacy on platelet response. Outcomes suggested that less frequent dosing can achieve adequate clinical response while increasing compliance, minimizing toxicity, and reducing cost.

The utilization of TPO receptor agonists as a long-term treatment for chronic ITP is a common practice. Despite evidence demonstrating remission in a minority of patients treated via this method, the majority of patients require ongoing maintenance therapy. Eltrombopag displays a plasma elimination half-life of 21-32 hours in healthy individuals and 26-35 hours in those with ITP [8]. This feature allows for less frequent dosing, potentially reducing the burden for patients. According to the case report, it has been demonstrated that the use of intermittent dosing has proven to be highly effective in achieving an optimal platelet response while avoiding any major bleeding or thrombotic events. Furthermore, the case report found that the requirement for rescue treatment was comparable to the findings of previous studies that examined daily dosing. The prescribing information for eltrombopag recommends an initial daily dose of 50 mg or 25 mg for patients of East Asian ethnicity. Subsequent dosage adjustments are based on platelet response with the objective of achieving a platelet count between 50,000 and 200,000 [9]. An alternate dosing protocol, however, proposes initiating treatment with eltrombopag 50 mg twice weekly or 25 mg daily for East Asian patients to achieve a minimum goal platelet count of 150,000. This approach may be considered in cases where rapid platelet count recovery is not critical or when the cost of treatment is a concern [9,10].

Undeniably, an alternative dosing protocol could lead to substantial cost savings. Intermittent dosing with a higher pill strength is likely to be a more cost-effective option than daily dosing in certain patients. While the present study did not allow for direct comparisons, a prospective controlled study must be conducted to explore this further. Such a study would provide critical insight into the efficacy and cost-effectiveness of this dosing approach. It would identify patient populations that stand to benefit most from this treatment plan. It is imperative that we pursue this line of inquiry to achieve better health outcomes and cost savings for patients [11].

The current algorithm used for daily dosing of eltrombopag in immune thrombocytopenia may not be suitable for all patients and may result in reduced adherence due to patient inconvenience. However, for individuals with chronic ITP who require TPO receptor agonist therapy, our research has indicated that an alternate intermittent powered eltrombopag dosing protocol is highly effective in maintaining an adequate platelet count [12]. This approach could offer a more personalized and efficient treatment option for patients with ITP.

## Conclusions

Standard approaches to treatment should constantly be reevaluated and modified when appropriate. Physicians should aim to reduce adverse drug effects and minimize pill burden and financial strain on patients while preserving treatment benefits. It may be useful to conduct additional studies comparing our proposed eltrombopag dosing protocol with the standard daily protocol. Such studies could help confirm our approach's clinical and cost-effectiveness, providing valuable insights that could inform future treatment protocols.
